# CSF Levels of Angiopoietin-2 Do Not Differ between Patients with CSF Fluid Leakage Syndrome and Controls

**DOI:** 10.1155/2015/343818

**Published:** 2015-09-10

**Authors:** Refik Pul, Özlem Yildiz, Franco Morbiducci, Thomas Skripuletz, Philipp Schwenkenbecher, Martin Stangel, Friedrich Götz, Georg Berding, Corinna Trebst, Frank Donnerstag

**Affiliations:** ^1^Department of Neurology, Hannover Medical School, Carl-Neuberg-Straße 1, 30625 Hannover, Germany; ^2^Department of Neuroradiology, Hannover Medical School, Carl-Neuberg-Straße 1, 30625 Hannover, Germany; ^3^Department of Nuclear Medicine, Hannover Medical School, Carl-Neuberg-Straße 1, 30625 Hannover, Germany

## Abstract

CSF abnormalities have been reported in CSF leakage syndrome. However, the mechanism for these CSF changes is actually unknown and they may indicate impaired CSF flow or blood-CSF barrier. Angiopoietin-2 (Ang-2), a protein which is expressed and released by endothelial cells, has been associated with increased vascular permeability. In the assumption that CSF changes are due to an impaired blood-CSF barrier, we hypothesized that subjects with persistent CSF leakage may have increased CSF Ang-2 levels. We enrolled 10 subjects with a clinically definite diagnosis of persisting CSF leakage syndrome and 10 control subjects. In CSF analyses, CSF to serum albumin ratio (Qalb) was the most frequently increased parameter indicating a disturbed blood-CSF barrier function. Comparison of the mean CSF Ang-2 levels, CSF to serum Ang-2 ratio (QAng-2), and QAng-2/Qalb between the control and CSF leakage patients did not show any significant difference. We suggest that the increase of Qalb results from a low CSF flow. Future studies with phase contrast-MRI in conjunction with CSF analyses before and after epidural blood patch treatment are required to address this question. It would be of particular interest whether Qalb can be used as a marker for successful nontargeted epidural blood patch treatment.

## 1. Introduction

Persistent cerebrospinal fluid (CSF) leakage syndrome is a rare cause of chronic headache. The common clinical presentation is orthostatic headache of dull aching nature, which, according to the International Headache Society Classification, occurs or worsens in less than 15 minutes after assuming the upright position and disappears or improves in less than 30 minutes after recumbency [1]. While secondary CSF loss can be an aftermath of a trauma, neurosurgical procedure, or even systemic dehydration, the etiology of spontaneous (primary) CSF loss remains unknown. Suspected factors are an underlying weakness of the meningeal sac/diverticula in certain regions and a trivial trauma [1]. The concept of such “loci minoris resistentiae” is supported by the fact that generalized connective tissue disorders predispose to the formation of dural defects allowing CSF leakage into the epidural or subdural space [1]. These leakages most often occur at the level of the thoracic spine or cervicothoracic junction but rarely at the skull base [2]. Elevated leukocyte counts and protein concentrations in the CSF have been reported in several cases and were attributed to hydrostatic CSF pressure changes [1, 3]. The exact mechanism of these CSF changes is not well understood and they may indicate alterations in the CSF flow or blood-CSF barrier. The blood-CSF barrier is constituted by several barriers and one of them is the choroid plexus (CP), a highly vascularized endothelial-epithelial convolute in the ventricular system and the main source of CSF production [4]. Epithelial cells of the CP are interconnected via dense tight junction strands which shield the CSF from the blood. In contrast, endothelial cells embedded in the extracellular matrix of the CP are constitutively fenestrated [4]. This fenestrated phenotype has been shown to be maintained by vascular endothelial growth factor (VEGF) [5, 6]. A gatekeeper of VEGF function is angiopoietin-2 (Ang-2) and has been shown to breach the endothelial barrier function by counteracting the inhibitory effect of Ang-1 [7]. Ang-2 has been reported to be expressed in endothelial cells of the mouse and buffalo CP suggesting that Ang-2 together with Ang-1 and VEGF is involved in the regulation of CSF production [4, 8, 9]. Ang-2 has also been shown to be expressed by ependymal cells which, in contrast to epithelial cells of the CP, form a less tight “cellular barrier” between CSF and brain parenchyma [4, 10]. Interestingly, increased levels of CSF Ang-2 have been reported in hypoxemic patients or those with traumatic brain injury/subarachnoid hemorrhage suggesting that these resulted by disruption/increased permeability of the blood-CSF or CSF-brain parenchyma barrier [11–13]. Less is known about Ang-2 in pathological conditions affecting the CSF compartment. We hypothesized that subjects with persistent CSF leakage may have altered CSF Ang-2 levels which may indicate an impaired or more permeable blood-CSF barrier, respectively.

## 2. Materials and Methods

In this study, 10 (8 female and 2 male) subjects with a clinically definite diagnosis of persisting CSF leakage syndrome were enrolled from February 2009 to April 2011 (http://www.ihs-classification.org/en/02_klassifikation/03_teil2/07.02.03_nonvascular.html). Beside the clinical symptoms, diagnosis was based on the findings in intrathecal gadolinium- (gd-) enhanced magnet resonance (MR) myelography and/or CSF scintigraphy (Table 1). For gd-enhanced MR myelography, gd (gadoterate meglumine, Dotarem, Guerbet, Villepinte, France) was injected intrathecally after obtaining informed consent. Subjects received a single dose of 0.2 mmol gd (≈0.4 mL 0.5 mmol/mL Gd-DOTA) and were maintained in a position of 35° head elevation. Conventional spin-echo sagittal and axial T1-weighted fat-suppressed sequences on a 1.5 T MR unit using a 35 mT/m gradient magnet (Siemens, Erlangen, Germany) enabled the detection of the site of the dural dehiscence. CSF scintigraphy was performed by administering 20 MBq (4.6 mCi) 111 indium-diethylene-triamine-pentaacetic acid via lumbar puncture. Images were obtained at 1 h and 4 h including the region of the urinary bladder and the area of lumbar puncture (posterior projection) after radioisotope injection. Early appearance of urinary bladder on the 4-hour image was noted (Table 1). At 24 and 48 h, images showed the activity distribution in the entire spinal subarachnoid space (dorsal projection) and the head (anterior, posterior, and both lateral projections). Images were obtained using large-field-of-view gamma cameras (DIACAM or ECAM, Siemens, Erlangen, Germany) equipped with medium-energy collimators. CSF and serum analyses were analyzed by standard methods before intrathecal administration of gd (Table 1) [14]. Ang-2 was measured in serum and CSF using the RayBio Human Ang-2 Elisa-Kit (Ray Biotech, distributed by Hoelzel Diagnostika GmbH, Köln, Germany) according to the manufacturer's instructions. All conditions were tested in duplicate for each independent measurement. The study was approved by the Institutional Review Board of the Hannover Medical School. All participants signed an informed consent form detailing the purpose of this study, the tests included in the exploratory protocol, and the permission to revoke the obtained data. Statistical analysis was performed using GraphPad Prism version 5.02. Normality of data was assessed by the Shapiro-Wilk test. Since the control cohort did not show a normal distribution of data, we performed the two-tailed Mann-Whitney *U* test to compare the mean Ang-2 values between the two cohorts. To analyze correlations to clinical measures two-tailed Pearson or Spearmen correlation coefficients were calculated. For each comparison, a *p* value < 0.05 was considered as statistically significant.

## 3. Results

Demographic and clinical characteristics of the enrolled subjects with persistent CSF leakage and those of controls are summarized in Table 1. Control subjects were recruited from an inpatient population admitted to the hospital with different neurological diagnoses. They were on average five years older (49 ± standard deviation (SD) 15 years) than subjects with CSF leakage (44 ± SD 13 years) and the proportion of females outweighed that of males in both groups. In general, patients with CSF leakage had a normal or almost normal body weight (mean body mass index (BMI) 22.1 ± SD 3.5), while the mean BMI of control subjects was higher (26.4 ± 6.7). We included this parameter since it has been shown to correlate positively with the CSF to serum albumin ratio, also referred to as Qalb, and a relationship with the epidural fat storage has been suggested [15]. Considering all participating subjects, we did not find any significant correlation of BMI to Qalb (Spearman *r* = 0.19, *p* = 0.413). Exclusion of one outlier (CSF leakage subject number 5 with Qalb 48.5) did not result in a significant correlation either (Spearman *r* = 0.39, *p* = 0.096).

**Figure 1:**
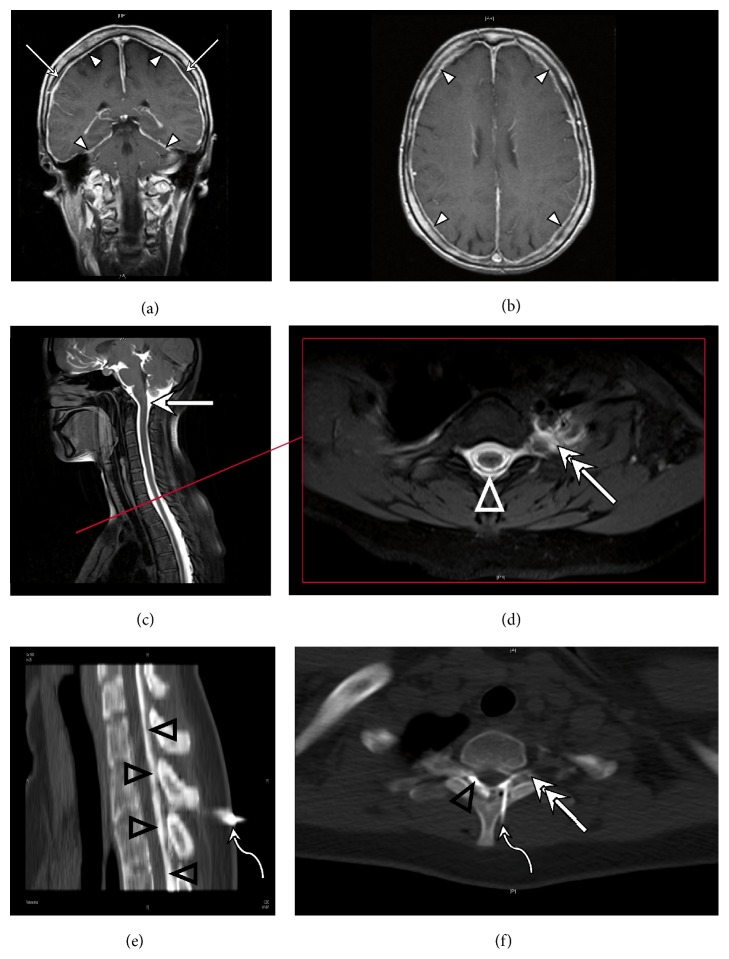
Imaging in a patient with symptomatic CSF leakage. (a + b) MRI with intravenous contrast media displays enhancement of the meninges over the convexity and tentorium (arrow heads). A fluid collection separates the periosteal and the inner layer of the enhancing dura mater (arrows). (c + d) MRI with intrathecally applied gadolinium contrast agent. (c) Distribution of contrast agent in the subarachnoid space (solid arrow). (d) MRI depicts transforaminal contrast leakage as the diagnosis of meningeal tears in the left T2 nerve root (double arrow) with epidural distribution of contrast agent in the epidural space (triangle). (e + f) CT-guided blood patch. (e) Tuohy needle in the epidural space of T1/2 (curved arrows) used to inject a mixture of blood and iodine contrast agent into the epidural space (black triangles). (f) Epidural blood patch by means of a Tuohy needle (curved arrow) with epidural (triangle) and transforaminal (double arrow) distribution of the blood at the level of the T2 nerve root.

Figure 1 shows characteristic findings in cranial MRI as well as the diagnostic procedure with intrathecal gd MR myelography (Figure 1). CSF scintigraphy resulted in the localization of the level of CSF leakages in 3 of 6 subjects, while MR myelography with intrathecal gd was more efficient with 9 of 9 subjects (Table 1). The early appearance of the radioisotope in the bladder enabled the diagnosis of CSF leakage in 5 of 6 subjects. Moreover, intrathecal gd MR myelography revealed additional CSF leakages in 3 subjects who had CSF leakages already detected by scintigraphy. Cranial MRI demonstrated subdural effusions in 9 of 10 subjects, while after the application of gd contrast agent meningeal enhancement was found in 7 of 9 subjects suggesting that subdural effusions are at least as reliable sign as meningeal contrast enhancement (Table 1).

Orthostatic headache, which mostly occurred occipitally, was a constant and most reliable symptom in all CSF leakage subjects. Despite the typical orthostatic nature of headache, time from onset of complaints to diagnosis varied considerably (mean time 102 ± 123 days, expressed as disease duration in Table 1) and in only two cases a traumatic event or a trigger was identified. In one of these subjects, CSF leakage was clearly attributed to a decompression surgery due to disc herniation at the level of C5/6 and fenestration of a spinal arachnoid cyst at the level of T2 to T4. Concerning relevant comorbidities, one subject in the CSF leakage cohort had Marfan's disease as a predisposing factor for CSF leakage (Table 1).

CSF analyses revealed lymphocytic pleocytosis in 4 cases with a cell count up to 23 leukocytes per *μ*L and CSF total protein was increased in 7 cases (normal range < 0.5 g/L). Qalb is a generally accepted measure of the blood-CSF barrier function and was increased in 7 subjects with CSF leakage [16]. In the control cohort, no subject revealed a CSF pleocytosis and the CSF total protein and Qalb were increased only in 4 and, respectively, in 3 cases. In each cohort, one subject exhibited type 2 oligoclonal bands, while in one subject of the CSF leakage cohort type 3 oligoclonal bands were detected. In none of the subjects, intrathecal immunoglobulin (Ig) G, IgA, or IgM synthesis was determined (Table 2).

The mean CSF Ang-2 concentration of all subjects was 0.31 ± SD 0.15 pg/mL without any difference between control and CSF leakage subjects (control 0.27 ± SD 0.09 pg/mL versus CSF leakage subjects 0.35 ± SD 0.20 pg/mL, *p* = 0.272, *U* = 35.0; Tables 2 and 3). The mean serum Ang-2 concentration of all subjects (1.23 ± SD 0.84 pg/mL) was approximately four times higher than the mean CSF Ang-2 concentration and again we did not detect any difference between the control and CSF leakage subjects (control 1.01 ± SD 0.49 pg/mL versus CSF leakage subjects 1.46 ± SD 1.10 pg/mL, *p* = 0.405, *U* = 38.5; Tables 2 and 3). The comparison of the CSF to serum ratio multiplied by 10^3^ of Ang-2 (expressed as QAng-2 in Tables 2 and 3) did not result in any statistical difference (control 313.0 ± SD 138.9 versus CSF leakage subjects 340.9 ± SD 249.8, *p* = 0.791, *U* = 46.0). Normalizing of QAng-2 by dividing by Qalb (QAng-2/Qalb) did not yield a statistical difference either (control 51.74 ± SD 32.19 versus CSF leakage subjects 38.52 ± SD 48.15, *p* = 0.105, *U* = 28.0; Tables 2 and 3). Moreover, there were no significant correlations of QAng-2 (Spearman *r* = −0.14, *p* = 0.565) and CSF Ang-2 concentrations (Spearman *r* = −0.05, *p* = 0.848) with Qalb. CSF IgG, IgA, and IgM concentrations and CSF albumin did not correlate with CSF Ang-2 concentrations or QAng-2 either (data not shown).

We did not detect any difference in mean CSF lactate concentrations between the two cohorts (control 1.8 ± SD 0.5 mmol/L versus CSF leakage subjects 1.7 ± SD 0.2 mmol/L, *p* = 0.625, *U* = 29.5; Tables 2 and 3). However, we found significant negative correlations for QAng-2 (Spearman *r* = −0.66, *p* = 0.003) and QAng-2/Qalb (Spearman *r* = −0.49, *p* = 0.045) with CSF lactate concentrations, while there was no significant correlation for CSF Ang-2 with CSF lactate concentration (Spearman *r* = 0.17, *p* = 0.520). There were no significant relationships when cell count was correlated with CSF Ang-2 concentrations, QAng-2, or QAng-2/Qalb (Spearman *r* ranged from −0.13 to 0.33; *p* ranged from 0.331 to 0.876). Furthermore, we could not find any association of Ang-2 with the obtained clinical or radiologic parameters.

## 4. Discussion

Our rationale for initiating this study was the observation of increased Qalb in several subjects with CSF leakage that attended our clinic. Qalb is generally used to evaluate blood-CSF barrier function and was indeed more frequently increased in our CSF leakage cohort. Albumin (66–69 kDa) is the most abundant protein in CSF comprising up to 67% of total CSF proteins and exclusively originates from blood [16, 17]. To reach CSF, albumin must cross the blood-brain and blood-CSF barrier [16, 17]. However, it is speculated that albumin is not able to pass the blood-brain barrier because of the presence of tight junctions and needs to be extracted from the plasma via fenestrated endothelial cells and to enter the CSF by crossing the epithelial cells of the CP via transcellular transport [18]. There is also speculation that the crossing of the epithelial cells is a passive process and may depend on the size of the CP [17]. However, there is also the view that all molecules are able to pass the blood-brain barrier and that the extent of protein transfer depends on the molecular size-dependent diffusion [16]. Moreover, caution must be taken in interpreting the increase of Qalb regarding the integrity of the brain barriers since elevation of Qalb can also be construed as a decline in the CSF secretion rate/turnover [16].

Ang-2 forms multimeric structures composed of monomers of 55 kDa and the native form is mainly present as disulfide-linked dimers, but variable oligomeric forms may exist as well [19]. So far, it remains unknown whether the CSF Ang-2 is an extraction from plasma, secreted by ependymal cells, or both. There is increasing evidence that Ang-2 plays a role in various conditions of plasma leakage by loosening interendothelial junctions [7, 20–22]. This activity at the “vessel wall” prompted us to hypothesize that the increase of Qalb is due to higher permeability of the blood-brain barrier and may explain increased Qalb values in subjects with CSF leakage syndrome. Consequently, we examined Ang-2 concentrations in serum and CSF of subjects with clinically definite and persistent CSF leakage syndrome. We found neither any difference in the mean CSF Ang-2, QAng-2, and QAng-2/Qalb as compared to our control cohort nor any correlation/association with the obtained CSF, clinical, or radiological parameters. In our study, the Ang-2 concentrations assessed in the CSF and serum concentrations are quite low suggesting quiescent ependymal or endothelial cells. The CSF Ang-2 levels of patients who suffered from subarachnoid hemorrhage have been reported to be 2.7-fold higher than control subjects, while only slightly elevated CSF Ang-2 concentrations have been observed in hypoxemic patients [12, 13]. In hypoxemic subjects, lactate that is abundantly released upon hypoxemia and known to activate receptor tyrosine kinases Axl, Tie2, and VEGF receptor 2 may have induced the release of Ang-2 as a compensatory response to hypoxia [23].

Among other factors, biomechanical stimulation has been shown to be a reliable trigger for the release of Ang-2 [24]. This is of particular interest since VEGF, whose function is mediated in corporation with Ang-2, has been implicated in the pathogenesis of hydrocephalus [25]. Accordingly, shear stress on arterial wall due to altered CSF flow or hemodynamics is considered as a possible trigger [26]. Since CSF leakage or, as a sort of biomechanical stimulation, pressure changes due to CSF leakage apparently do not lead to an increased release of Ang-2 into the CSF, the question of how and why Qalb increases in CSF leakage syndrome arises. In this regard, it should be mentioned that subjects with CSF leakage syndrome have been shown to exhibit a lower CSF flow as demonstrated by phase contrast-MRI [27]. We suggest that this is most probably the cause for the increase in Qalb which is supported by the fact that the concentrations of IgG, IgA, and IgM show that the molecular size-related selectivity of the barrier function is maintained in spite of blood-CSF barrier dysfunction (data not shown). Thus, the entry of plasma proteins via the leakage in CSF leakage syndrome seems rather unlikely. It should be mentioned that all CSF leakage subjects received an epidural blood patch treatment and intrathecal gd MR myelography provided a successful targeted approach in 9 of these subjects. The technique of intrathecal gd MR myelography is only available in specialized centers, while the epidural blood patch treatment is more common and mostly done in a nontargeted manner. It remains elusive whether Qalb can be used as a marker for successful nontargeted blood patching. Certainly, a constraining factor in this context is that CSF analyses are increasingly omitted since lumbar punctures are considered as a potential new source for a leakage. Further studies which will include CSF analyses and phase contrast-MRI before and after epidural blood patch treatment may shed light on the origin of the observed CSF changes in CSF leakage syndrome and whether Qalb can be used as a treatment marker.

Ang-2 and related molecules are of high interest for the CSF compartment and may help to elucidate our understanding of the mechanism of the brain barriers and CSF hemodynamics. In this pilot study, we did not find any involvement of Ang-2 in CSF leakage syndrome. There are several limitations inherent in this study. One limitation concerns the exploratory analysis of correlations which are post hoc exploratory analyses that need validation from independent studies. The second limitation is that the control group consisted of subjects who were not healthy. Therefore, a difference in CSF Ang-2 levels between healthy controls and subjects with CSF leakage syndrome cannot be excluded. The control group was deliberately chosen in order to find out whether other conditions, which may lead to an increase of Qalb, will be also accompanied by changes in CSF Ang-2 levels. Unexpectedly, we did not find any difference in CSF Ang-2 levels despite the significantly higher Qalb values and ratios of immunoglobulins in subjects with CSF leakage syndrome suggesting that secretion and elimination of Ang-2 are different from these proteins. Further limitations of the study are the small number of enrolled patients and that we assessed only a single marker of endothelial activation.

## Figures and Tables

**(a):** 

Subjects	Demographic and clinical characteristics of subjects with CSF leakage								
	Age (years)	Gender	BMI	Relevant comorbidities	Symptoms	Orthostatic symptoms	DD (days)	Prior traumatic event	Other events before disease onset
1	50	f	24.7	None	Headache left frontal	Yes	30	No	Physical exertion
									
2	40	f	18.4	Marfan's syndrome, Arnold-Chiari I malformation, and depressive disorder	Occipital and temporal headache	Yes	14	No	No
									
3	43	m	26	None	Occipital headache	Yes	10	No	No
									
4	45	f	23.7	Migraine with aura	Occipital headache, pressure feeling in left ear, diplopia, and paresthesia in left arm and leg	Yes	173	No	No
									
5	72	f	14.7	Subtotal colectomy, short bowel syndrome, cachexia, chronic gastritis, and numerous intestinal operations due to recurrent ileus	Occipital headache, vertigo	Yes	360	No	No
									
6	30	f	21.5	None	Holocephalic headache	Yes	30	No	No
									
7	44	m	25.2	Arterial hypertension	Holocephalic headache, vertigo, vomiting, diplopia, and paresthesia in left arm and leg	Yes	196	Fenestration of a spinal arachnoid cyst T2–4	Decompression surgery due to disc herniation at the level of C5/6
									
8	23	f	22.8	None	Occipital headache, vertigo, and photosensitivity	Yes	4	No	No
									
9	44	f	20.2	None	Occipital headache, vertigo, vomiting, and abducens nerve palsy	Yes	2	No	No
									
10	52	f	23.4	Cervical conisation due to cervix carcinoma and decompression surgery due to disc herniation at the level of L5/S1	Holocephalic headache	Protracted	202	No	No

**(b):** 

Subjects	Radiological characteristics of subjects with CSF leakage					
	Method of detection	Level of leakage	CSF scintigraphy	Early detection (in the bladder)	Meningeal enhancement (cranial MRI)	Subdural effusions (cranial MRI)
1	Intrathecal gadolinium enhanced MR myelography	C1/2, T12	Not detected	Yes	Yes	Yes
2	Intrathecal gadolinium enhanced MR myelography	L1/2, L2/3	n.d.	n.a.	n.d.	Yes
3	Intrathecal gadolinium enhanced MR myelography	C7/T1, T1/2	C7/T1	Yes	Yes	Yes
4	Intrathecal gadolinium enhanced MR myelography	C6/7, T1/2, T11, 12, L1/2, L3/4	L1/2, L3/4, T11/12	Yes	No	No
5	Intrathecal gadolinium enhanced MR myelography	C5/6, C6/7 (nerve root cysts L4/5, S2/3, S3)	Not detected	Yes	Yes	Yes
6	Intrathecal gadolinium enhanced MR myelography	C1/2, C5/6, C6/7, T12/L1	n.d.	n.a.	Yes	Yes
7	Intrathecal gadolinium enhanced MR myelography	(Weak) C4/5, T1/2	Not detected	Yes	No	Yes
8	Intrathecal gadolinium enhanced MR myelography	C7/T1, T1/2, T2/3	n.d.	n.a.	Yes	Yes
9	Intrathecal gadolinium enhanced MR myelography	C6/7, T1/2	n.d.	n.a.	Yes	Yes
10	MRI of the spine	Not detected (multiple nerve root cysts, 10 mm T1/2 and 25 mm S1/2)	S1/2	No	Yes	Yes

**(c):** 

Controls	Demographic and clinical characteristics of control subjects				
	Age (years)	Gender	BMI	Diagnosis	Relevant comorbidities
1	74	f	20.6	Axonal polyneuropathy	Arterial hypertension, atrial fibrillation
2	45	m	26.6	Essential tremor	Alcohol dependence, recurrent depressive disorder
3	48	m	27.2	Multifocal motor neuropathy	Atopic dermatitis
4	55	f	25.7	Cervical vertigo	Disc herniation at the level of T6/7, asthma
5	65	m	31.2	Demyelinating neuropathy	Arterial hypertension, heart failure, psoriasis, gout
6	45	f	42.0	Axonal polyneuropathy	Arterial hypertension, diabetes mellitus, asthma, and anxiety disorder
7	48	f	19.2	Somatic symptom disorder	Depressive disorder, anxiety disorder
8	31	f	28.7	Axonal polyneuropathy	Borderline personality disorder
9	24	f	20.5	Somatic symptom disorder	None
10	59	m	22.5	Motor neuron disease	None

**Table 2:** Overview of cerebrospinal fluid (CSF) and angiopoietin-2 (Ang-2) parameters. Q: quotient, that is, CSF to serum ratio; alb.: albumin; Ig: immunoglobulin; C: control subjects; LS: subjects with CSF leakage; LP: lymphocytic pleocytosis; N: normal; ABC: artificial blood contamination; n.a.: not applicable; and n.d.: not done.

	CSF characteristics										Ang-2	
	Cell count per *µ*L	Cytology	CSF total protein (mg/L)	Qalb (CSF/serum × 10^3^)	Blood-CSF barrier function	IgG synthesis	IgA synthesis	IgM synthesis	Oligoclonal bands	Lactate (mmol/L)	QAng-2 (CSF/serum × 10^3^)	QAng-2/Qalb
Subjects	LS											

1	15.3	LP	1110	13.2	Impaired	No	No	No	Type II	n.d.	106.8	8.1
2	23.0	LP	896	8.8	Impaired	No	No	No	Type I	1.8	193.0	21.9
3	15.0	LP	1021	18.7	Impaired	No	No	No	Type III	1.7	403.6	21.6
4	1.3	N	476	5.9	Intact	No	No	No	Type I	1.6	203.6	34.5
5	0.3	ABC	2900	48.5	n.a.	No	No	No	Type I	n.d.	46.6	0.96
6	6.7	LP	365	4.8	Intact	No	No	No	Type I	n.d.	803.9	167.5
7	3.0	N	1033	15.4	Impaired	No	No	No	Type I	1.5	584.4	38.0
8	0.3	ABC	1044	17	Impaired	n.a.	n.a.	n.a.	Type I	1.8	418.6	24.6
9	2.0	N	729	9.9	Impaired	No	No	No	Type I	2.0	105.9	10.7
10	3.0	N	562	9.5	Impaired	No	No	No	Type I	1.7	542.4	57.3

Controls	C											

1	0.7	N	404	5.3	Intact	No	No	No	Type I	1.7	218.3	41.5
2	1.0	N	413	6.3	Intact	No	No	No	Type I	1.9	235.8	37.7
3	2.3	N	796	14.8	Impaired	No	No	No	Type I	2.0	222.4	15.1
4	0.3	N	377	5.4	Intact	No	No	No	Type I	1.4	633.5	116.5
5	3.3	N	534	9.2	Impaired	No	No	No	Type II	1.7	254.4	27.5
6	0.3	N	654	10.1	Impaired	No	No	No	Type I	2.9	332.5	32.8
7	1.0	ABC	236	3.5	Intact	No	No	No	Type I	1.8	175.3	50.4
8	1.3	N	345	5.7	Intact	No	No	No	Type I	1.5	243.5	43.1
9	3.3	N	338	4.4	Intact	No	No	No	Type I	1.6	443.1	101.4
10	0.3	N	506	7.2	Intact	No	n.d.	No	Type I	1.3	371.0	51.5

**Table 3:** Summary of the statistics. CSF: cerebrospinal fluid; C: control subjects; LS: subjects with CSF leakage; Ang-2: angiopoietin-2; Q: quotient, that is, CSF to serum ratio; alb.: albumin; Ig: immunoglobulin.

	LS			C			*p*
	Median	25% percentile	75% percentile	Median	25% percentile	75% percentile	
Cell count	3.00	1.05	15.08	1.00	0.30	2.55	0.092
CSF total protein (g/L)	0.74	0.45	1.02	0.41	0.34	0.56	0.063
CSF lactate (mmol/L)	1.74	1.57	1.83	1.73	1.44	1.88	0.625

Qalb	11.55	8.08	17.43	5.96	5.04	9.47	0.028
Mean CSF Ang-2 (pg/mL)	0.33	0.21	0.43	0.25	0.22	0.31	0.272
Mean serum Ang-2 (pg/mL)	1.04	0.76	2.00	0.93	0.58	1.36	0.405
QAng-2	0.30	0.11	0.55	0.25	0.22	0.39	0.791
QAng-2/Qalb	23.3	10.1	42.8	42.3	31.5	63.9	0.105

IgG ratio	7.90	4.43	10.64	2.99	2.44	6.56	0.036
IgA ratio	4.40	2.66	7.07	1.77	1.32	2.98	0.013
IgM ratio	1.60	1.00	2.72	0.48	0.35	0.72	0.002
